# Tumor Invasion Distance Based on MRI Is a Novel Prognostic Indicator for I-IIIB Cervical Cancer Patients Treated with Radiotherapy

**DOI:** 10.3390/curroncol32060355

**Published:** 2025-06-16

**Authors:** Linying Liu, Jie Lin, Anyang Li, Ning Xie, Jianfeng Zheng, Youping Xiao, Xuefen Lin, Shizhong Wu, Haijuan Yu, Yang Sun

**Affiliations:** 1 Department of Gynecology, Clinical Oncology School of Fujian Medical University, Fujian Cancer Hospital, Fuma Road, Fuzhou 350014, China; 2 Department of Radiology, Clinical Oncology School of Fujian Medical University, Fujian Cancer Hospital, Fuzhou 350014, China

**Keywords:** cervical cancer, radiotherapy, tumor invasion distance, MRI, prognosis

## Abstract

**Aims:**This study aimed to identify the prognostic value of tumor invasion distance (TID) based on MRI findings in cervical-cancer (CC) patients treated with radiotherapy (RT). **Methods**: A total of 218 CC patients diagnosed at Fujian Cancer Hospital from December 2018 to December 2019 were included in the study. Cox regression analyses were conducted to identify independent prognostic factors for overall survival (OS), including low 1/3 vaginal involvement, a longer TID, and RT without chemotherapy. These factors were subsequently used to construct a nomogram for individualized risk prediction. Kaplan–Meier survival analysis was employed to evaluate survival outcomes and establish a risk stratification system. The performance of the new stratification was assessed using the linear trend χ^2^ test, Akaike information criterion, and Harrell’s concordance index. **Results**: A longer TID was associated with worse 3-year OS (*p* < 0.001, HR: 3.42, 95% CI: 1.67–7.00). A longer TID, lower 1/3 vaginal involvement, and concurrent chemotherapy were independent prognostic survival factors for CC patients. Compared with the 2018 FIGO staging system, the new risk stratification system provided better monotonicity with a higher linear trend χ^2^ value (28.03 vs. 9.35), better discriminatory ability with smaller Akaike information criterion (312 vs. 331), and a greater Harrell C statistic (0.74 vs. 0.65) for predicting 3-year OS. **Conclusions**: This was the first study to demonstrate the prognostic value of TID in CC patients who received RT. The new risk stratification system based on TID could complement the 2018 FIGO staging system in identifying high-risk patients for more intense treatment and care. Further prospective research with larger samples is warranted to confirm the significance of TID for CC patients treated with RT.

## 1. Introduction

Cervical cancer (CC) is one of the foremost common cancers in women [1], with an estimated 660,000 new cases and 350,000 deaths worldwide in 2022 [2]. The therapeutic approaches and prognosis are predominantly influenced by the tumor’s invasion, determined during initial diagnosis, underscoring the importance of accurate CC staging [3]. In the past, the staging of CC was based on the International Federation of Gynecology and Obstetrics (FIGO) staging system to ensure a globally standardized assessment [4]. However, it mainly relies on gynecological examination, which is inherently subjective and prone to biases [3,5].

In clinical practice, the accuracy of disease assessment has varying effects on the prognosis of CC depending on the chosen treatment modality. For early-stage CC treated with surgery, the Sedlis criteria define the risk factors for recurrence warranting and services as the adjuvant treatment guidance [6]. Despite differences between the preoperative and postoperative stages, patients could still receive relatively adequate treatment based on the Sedlis criteria [7,8]. Hence, the survival outcome of surgical patients could be guaranteed. However, the prognosis of CC patients who received radiotherapy (RT) has still been an intractable problem in recent years. Almost 40% of locally advanced patients suffered from disease recurrence after RT [9] and the reported 5-year overall survival rate is only 50–70% [10]. Unreliable disease assessment could misguide treatment strategies and lead to an unimproved prognosis. Therefore, it is urgently required that reliable factors are found for guiding treatment strategies and predicting the prognosis of CC patients who received RT. In 2018, FIGO revised the staging system by incorporating magnetic resonance imaging (MRI) for assessing the status of lymph node metastasis [11]. This perspective prompts consideration that local tumor invasion based on MRI could also serve as a prognostic factor for CC patients treated with RT.

Cervical tumor-growth patterns could predict the primary response to RT and prognosis [12,13,14]. Trimbos et al. showed that barrel-shaped bulky tumors (>4 cm) exhibited significantly worse OS compared with a small tumor (<4 cm) and other bulky exophytic tumors in CC [15]. In a clinical setting, we also observe cervical tumor eccentric growth with a longer invasion distance, which always implies more severe tumor adhesion to surrounding tissues, which increases surgery difficulties and worsens the prognosis. Moreover, according to the Sedlis criteria, the tumor-stromal invasion, to different degrees, impacts the supplementary treatment plan [16]. Another study also confirmed the tumor-stromal invasion was also an indicator of lymph node metastasis [17]. However, few studies have focused on the significance of eccentric growth with a longer invasion distance in CC treated with RT.

Therefore, in this study, we aimed to investigate the prognostic value of eccentric growth with a different invasion distance in CC patients treated with RT. Correspondingly, we first defined a novel concept of tumor invasion distance (TID) based on MRI and retrospectively studied the relationship between the TID and prognosis of CC patients. Additionally, we constructed a nomogram incorporating clinical features and MRI characteristics to predict the prognosis of CC patients. Finally, we compared our new risk category with the FIGO 2018 staging system to assist physicians in outcome prediction and with making and devising personalized treatment strategies for patients treated with RT.

## 2. Methods

### 2.1. Patients

This study is a retrospective analysis. The Ethics Committee of Fujian Cancer Hospital has reviewed and approved this study (K2024-085-01). Informed consent to participate was obtained from all of the participants in the study. The inclusion criteria were as follows: CC patients treated with radiotherapy with or without concurrent chemotherapy followed by brachytherapy at our institution from December 2018 to December 2019. FIGO 2018 was used to determine the cancer stage. The exclusion criteria were as follows: (1) non-initial diagnosed cases; (2) anti-tumor treatment history; (3) pelvic surgery history; (4) incomplete radiotherapy; (5) having received surgery treatment; (6) using non-intravenous or platinum-free chemotherapy; (7) lack of pre-treatment MRI; (8) loss of follow-up; and (9) IIIC-IVB. To explore the impact of local lesion characteristics on prognosis, we excluded CC patients in stages IIIC-IVA. The patient selection process is outlined in Supplementary Figure S1. The following data was collected: (1) clinical features, such as age, stage, pathology, and treatment, etc. and (2) MRI image characteristics, including vaginal involvement, TID, uterus involvement, and hydronephrosis.

### 2.2. Evaluation and Treatment

Patients underwent adequate pretreatment evaluations, including medical history, physical (gynecological) examination, hematological testing, computed tomography (CT) of the chest, abdominal ultrasonography or CT, and MRI of the pelvis, or PET-CT [18].

Treatment was mainly composed of external pelvic beam radiotherapy (EBRT) with intensity-modulated radiotherapy (IMRT) or the conventional 4 or 6 fields box conformal RT technique, followed by individualized high-dose-rate intracavitary brachytherapy (HDR-ICBT) with 192 Ir. The EBRT consists of 45–50 Gy/25–30 F, followed by the HDR-ICBT for 28.0 Gy/4 F. Cisplatin-based concurrent chemoradiotherapy (CCRT) was recommended unless infeasible. Cisplatin monotherapy (40 mg/m^2^) or combination therapy with cisplatin (60–75 mg/m^2^) or nedaplatin (80 mg/m^2^) plus paclitaxel (130–175 mg/m^2^) was administered every three weeks during RT.

### 2.3. Tumor Invasion Distance Measurement

**Figure 1:**
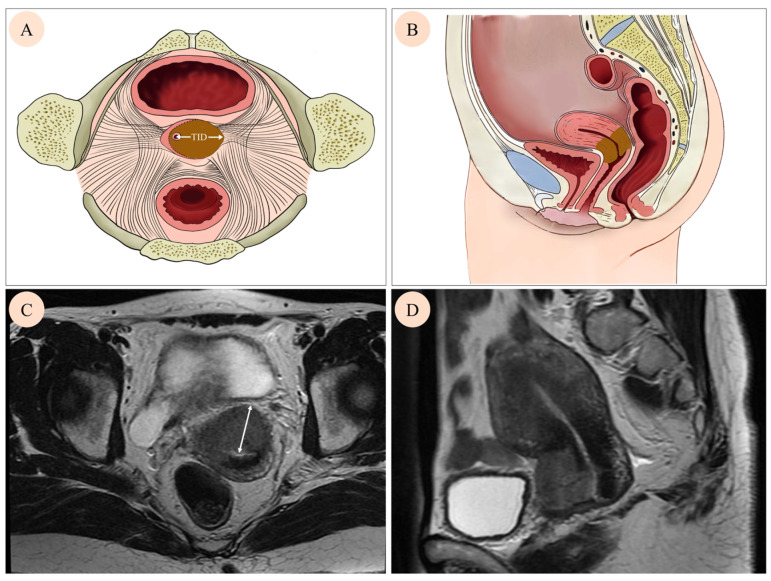
Cervical tumor growth on different images. A mass in the cervix was found by axial image (**A**), sagittal image (**B**), axial T2 weighted MRI image (**C**), and sagittal T2 weighted MRI (**D**). Tumor invasion distance (TID) was defined as the distance from the center of the cervical canal to the farthest edge (white arrow on (**A**,**C**)).

Pre-treatment pelvic MRI was conducted using a high-field MR scanner (1.5 T or 3.0 T) with a contrast injection. The imaging protocol comprised T1-weighted (T1W), T2-weighted (T2W), and Diffusion-weighted (DW) images on various planes. For each patient, the plane revealing the maximum invasion distance was selected, and the TID was defined as the distance from the center of the cervical canal to the farthest edge (Figure 1A,B). Corresponding schematic representations of TID on MRI images are shown in Figure 1C,D, illustrating how the measurement was applied in clinical imaging. Two experienced gynecological radiologists (GA and GB) measured the TID based on fused images of transverse or oblique axial T2W sequences.

### 2.4. Follow-Up and Outcomes

Follow-up was conducted from the day of diagnosis to the day of death or the day of the last follow-up. After treatment, patients underwent assessments every three months during the first two years, followed by semi-annual evaluations for the subsequent three to five years, and annual assessments after five years.

The primary outcome was overall survival (OS), defined as the interval from the diagnostic day to death or the last follow-up. The secondary outcomes included progression-free survival (PFS), locoregional relapse-free survival (LRFS), and distant metastasis-free survival (DMFS). PFS was defined as the interval from the diagnostic day to the onset of regional recurrence, distant metastasis, death, or the last follow-up. LRFS was defined as the interval from the diagnostic day to the locoregional progression, the last follow-up, or death. DMFS was defined as the interval from the diagnostic day to the distant metastasis, the last follow-up, or death. The last follow-up was in February 2023.

### 2.5. A New Risk Stratification System

#### 2.5.1. The Prognostic Value of TID and a New Risk Stratification

Firstly, the prognostic values of TID were assessed in terms of OS, PFS, LRFS, and DMFS. Subsequently, univariate and multivariate Cox regression analyses were performed to identify independent prognostic factors. The identified independent factors were then incorporated into a prognostic model construction. Evaluation of the nomogram’s discrimination and calibration ability was carried out using the C-index and calibration curves, respectively.

After the identification of independent prognostic factors, each factor was assigned a score by delineating vertical lines on the respective axes of each covariate. Each patient was allocated an individual risk score by summing up their relevant factors. Finally, patients were stratified into low-, middle-, and high-risk groups according to the optimal value of the total scores.

#### 2.5.2. The Comparison of New Risk Stratification and FIGO Staging System

After adjusting clinical factors, the Cox proportional hazards regression model was used to compare the 3-year OS rates between new risk stratification and the 2018 FIGO staging system. Monotonicity was assessed using the linear trend χ^2^ test, where a higher value indicated a more favorable monotonic trend. The discriminability of a gradient evaluation for the two models was appraised using the Akaike information criterion (AIC) and Harrell C statistic. A model with a lower AIC was favored to mitigate the risk of overfitting. Furthermore, a detailed analysis of secondary cumulative 3-year outcomes (PFS, LRFS, and DMFS) was conducted for the newly proposed stratification system.

### 2.6. Statistical Analysis

All statistical analyses were performed using SPSS (version 26.0) and R (version 4.0.2). The optimal cutoff values for the continuous variables were determined using the X-tile application (https://medicine.yale.edu/lab/rimm/research/software/, accessed on 10 January 2023). Kaplan–Meier survival analysis was employed to assess survival outcomes, and group differences were evaluated using the log-rank test. Median follow-up time was calculated using the reverse Kaplan–Meier method to account for censoring. Cox proportional hazards regression models were applied for univariate and multivariate survival analyses to identify independent prognostic factors. Based on these factors, a nomogram was constructed using the rms package, while the timeROC package was used to calculate the time-dependent area under the curve (AUC). Calibration curves generated via the rms package assessed the agreement between the predicted and observed outcomes. Decision curve analysis (DCA) was conducted using the ggDCA package to compare the clinical net benefit of different prognostic models. The nomogram’s discriminative ability was evaluated using the concordance index (C-index). The Shapiro–Wilk test assessed data normality, and Spearman’s correlation test evaluated the association between TID and tumor size. All of the reported *p* values were two-sided, with statistical significance set at *p* < 0.05.

## 3. Results

### 3.1. Cohort Characteristics and Survival

A total of 988 CC patients who underwent RT at our institution between December 2018 and December 2019 were initially screened. After applying the exclusion criteria, 218 eligible patients were ultimately included in the final analysis. As shown in Table 1, the median age was 59 years (range: 25–84 years). There were 1.4% stage I patients (*n* = 3), 50.9% stage II (*n* = 111), and 47.7% stage III (*n* = 104). Among all of the participants, 72.9% (*n* = 159) were treated with CCRT and 27.1% (*n* = 59) were treated with RT alone. Optimal cutoff values were determined for age (73 years) and TID (3.9 cm), respectively.

Median follow-up time was 42 months (range 3–49 months). In total, 15.6% (*n* = 34) patients died, 23.9% (*n* = 52) suffered from disease progression, 8.3% (*n* = 18) patients experienced locoregional recurrence, and 14.2% (*n* = 31) developed distant metastasis at their last follow-up. The estimated 3-year OS, PFS, LRFS, and DMFS rates were 85.3%, 76.6%, 91.7%, and 85.8%, respectively.

### 3.2. The Relationship Between TID and Outcomes of CC Patients

**Figure 2:**
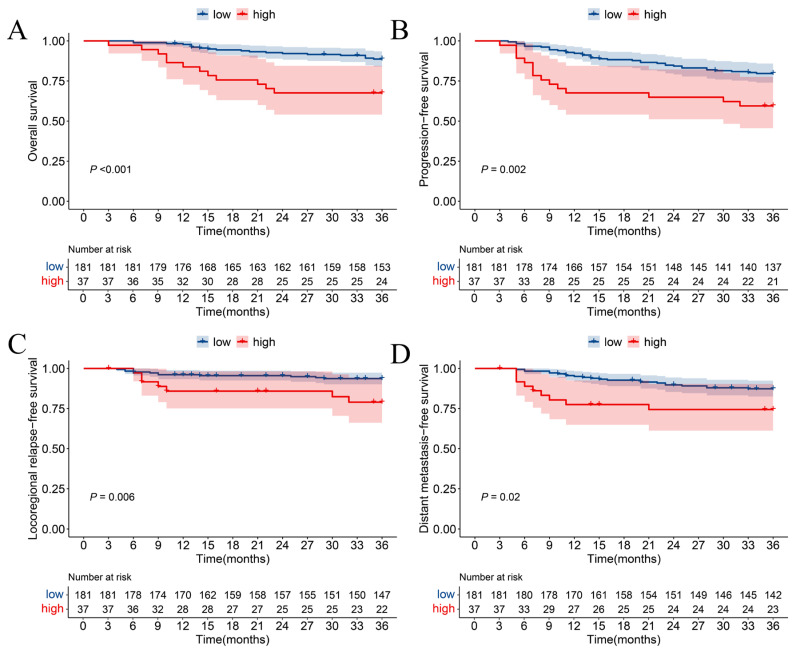
Kaplan–Meier survival curves of (**A**) overall survival, (**B**) progression-free survival, (**C**) locoregional relapse-free survival, and (**D**) distant metastasis-free survival in different degrees of TID.

According to survival analysis, compared with the low group, the high-level TID group had worse OS as shown in Figure 2A (*p* < 0.001, Hazard Ratio [HR]: 3.419, 95% Confidence Interval [CI]: 1.671–6.998), worse PFS shown in Figure 2B (*p* = 0.002, HR: 2.488, 95% CI: 1.361–4.545), worse LRFS shown in Figure 2C (*p* = 0.006, HR: 3.5, 95% CI: 1.356–9.036), and worse DMFS shown in Figure 2D (*p* = 0.02, HR: 2.446, 95% CI: 1.126–5.315).

### 3.3. The Relationship Between TID and Tumor Size

To evaluate if the TID was associated with tumor size, a correlation test was conducted. A significantly positive correlation was found between TID and size (*p* = 0.019, Spearman r 0.721).

### 3.4. Identification of Prognostic Factors

Univariate and multivariate Cox regression models for predictors of OS, PFS, DMFS, and LRFS are shown in Table 2. Multivariate analysis revealed that a longer TID was the independent risk factor for OS (HR: 3, 95% CI: 1.41–6.38, *p* = 0.004), PFS (HR: 2.37, 95% CI: 1.25–4.49, *p* = 0.008), DMFS (HR: 2.45, 95% CI: 1.12–5.32, *p* = 0.024), and LRFS (HR: 3.94, 95% CI: 1.52–10.24, *p* = 0.005). Low 1/3 vaginal involvement was significantly associated with low OS (HR: 2.58, 95% CI: 1.16–5.76, *p* = 0.021) and PFS (HR: 2.02, 95% CI: 1.01–4.04, *p* = 0.048). Conversely, CCRT was an independent protective factor for OS (HR: 0.32, 95% CI: 0.15–0.7, *p* = 0.004), PFS (HR: 0.41, 95% CI: 0.22–0.77, *p* = 0.005), and LRFS (HR: 0.29, 95% CI: 0.12–0.75, *p* = 0.01).

### 3.5. Establishment of a Prognostic Model and New Risk Stratification

**Figure 3:**
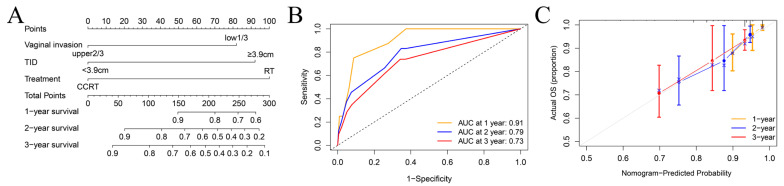
Construction and validation of the predictive model in CC patients. (**A**) Nomogram to estimate the 1-,2-,3-year survival possibility. (**B**) The ROC curves for the predicted 1-,2-, and 3-year survival rate. (**C**) The calibration curves for nomogram validation.

We established a prognostic model based on parameters including vaginal involvement, TID, and treatment (Figure 3A). The AUC for 1-, 2-, and 3-year OS were 0.91 (95% CI: 0.83–0.98), 0.79 (95% CI: 0.69–0.89), and 0.73 (95% CI: 0.63–0.83), respectively (Figure 3B). The calibration curves demonstrate the robust performance of the nomogram (Figure 3C). The C-index was 0.74.

This nomogram assigned individual scores (range: 0–274.1) by adding up risk factor points (lower 1/3 vaginal involvement: 81.9, longer TID: 92.2, RT: 100). Utilizing the cumulative score, the 3-year overall survival (OS) risk was stratified into three levels: low-risk group (0–81.9), middle-risk group (90–100), and high-risk group (>100).

### 3.6. Comparison of New Risk Stratification and the 2018 FIGO Stage System

**Figure 4:**
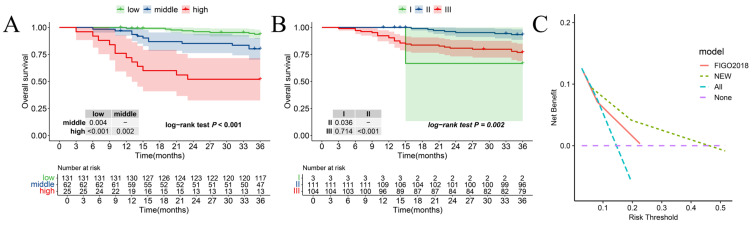
Comparing the new risk stratification and the 2018 FIGO stage system. Kaplan–Meier for 3-year OS according to (**A**) the new risk stratification and (**B**) the 2018 FIGO stage system. (**C**) The DCA of the new risk stratification and 2018 FIGO stage system.

The Kaplan–Meier curves regarding the new risk stratification and 2018 FIGO stage are shown in Figure 4A,B. Within the new stratification, 131 patients in the low-risk group exhibited a 3-year OS of 93.7% (95% CI: 0.90–0.98), 62 patients in the middle-risk category showed an 80.2% 3-year OS (95% CI: 0.71–0.91), while 25 patients categorized as high risk had a 52.0% 3-year OS (95% CI: 0.36–0.76). Notably, patients with higher risk levels experienced a significantly worse prognosis (*p*_low-risk vs. middle-risk_: 0.004, *p*_middle- vs. high-risk_: 0.002, *p*_low- vs. high-risk_: <0.001, *p*_overall_: <0.001). In comparison, the 2018 FIGO stage system exhibited a 93.3% 3-year OS (95% CI: 0.89–0.98) of patients with stage II and 76.9% a 3-year OS (95% CI: 0.69–0.85) with stage III. Contrasting with the 2018 FIGO stage system, the new risk stratification demonstrated superior monotonicity, as evidenced by a higher χ^2^ value for the linear trend (28.03 vs. 9.35). Additionally, the new stratification exhibited better discriminatory ability for 3-year OS, reflected in a smaller Akaike information criterion (AIC) value (312 vs. 331) and a greater C statistic (0.74 vs. 0.65) (Table 3). As shown in Figure 4C, the decision curve analysis showed that if the threshold probability ranges from 8 to 45%, employing the new risk stratification nomogram for predicting a 3-year overall survival (OS) yields greater benefits compared to the 2018 FIGO stage system.

### 3.7. The Relationship Between New Risk Stratification and Secondary Outcomes

**Figure 5:**
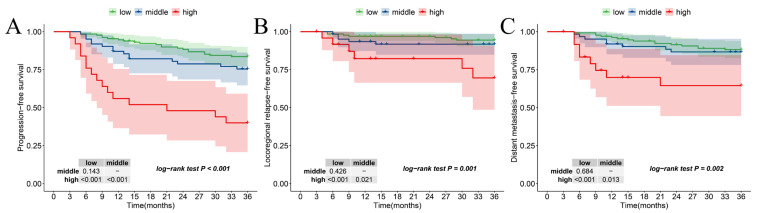
Kaplan–Meier according to the new risk stratification in (**A**) PFS, (**B**) LRFS, and (**C**) DMFS.

The Kaplan–Meier curves pertaining to the new risk stratification and other outcomes are shown in Figure 5. In comparison with the low- and middle-risk levels, patients classified in the high-risk level exhibited significantly worse PFS (*p*_middle-risk vs. high-risk_: <0.001, *p*_low-risk vs. high-risk_: <0.001, *p*_overall_: < 0.001) (Figure 5A), LRFS (*p*_middle-risk vs. high-risk_: <0.001, *p*_low-risk vs. high-risk_: =0.021, *p*_overall_: =0.001) (Figure 5B), and DMFS (*p*_middle-risk vs. high-risk_: <0.001, *p*_low-risk vs. high-risk_: <0.013, *p*_overall_: =0.002) (Figure 5C).

## 4. Discussion

In the present study, we explored the prognostic significance of tumor eccentric growth in CC treated with RT. Correspondingly, we first defined TID, an MRI-based indicator, to represent the severity of tumor eccentric growth. Tumor invasion distance was defined as the longest distance from the cervical canal to the edge of the tumor. Our analysis identified TID as a novel independent predictor for survival outcomes in CC patients undergoing RT. Based on TID, vaginal invasion, and the RT mode, we established a nomogram for outcomes in CC patients who underwent RT, which demonstrated excellent discrimination ability. Furthermore, according to our risk stratification, patients with high-risk scores had worse OS, PFS, LRFS, and DMFS, suggesting potential benefits from additional therapy and more rigorous follow-up plans. This new risk stratification has the potential to complement the 2018 FIGO stage, offering a personalized approach for treatment and surveillance optimization in CC patients who received RT.

The current pre-treatment assessment for CC primarily relies on physical examination, but pelvic inflammation may affect diagnosis [19], leading to inconsistencies in treatment strategies and prognosis. Ultrasound (US) is commonly used for initial screening due to its low cost and ease of operation, but its resolution is limited and highly dependent on operator experience [20]. In the evaluation of cervical cancer, ultrasound is less reliable than MRI in distinguishing inflammation from malignancy. Although increasing evidence suggests that the developed US can achieve similar diagnostic performance comparable to MRI, including assessing tumor size and parametrial invasion, it is only recommended for pre-treatment assessment of CC under resource-limited settings without MRI [21,22,23,24]. In contrast, MRI provides higher soft-tissue resolution, allowing the precise visualization of tumor extent, depth of invasion, and surrounding involvement [25], with more stable imaging results unaffected by operator variability [20]. Although discrepancies exist between MRI-based tumor involvement assessments and pathological findings [26,27], its noninvasive nature and practicality make it indispensable in pre-treatment evaluation. Currently, MRI is integrated into the modified FIGO system for CC assessment [28]. Our study demonstrates that MRI-based TID and vaginal involvement serve as prognostic predictors for CC patients undergoing radiotherapy, complementing the 2018 FIGO staging system.

In our study, tumor invasion distance based on MRI serves as a prognostic predictor for non-surgical CC patients. Post-surgically pathological “tumor stromal invasion” was identified as a prognostic indicator and a guiding factor for supplementary treatments in early-stage CC patients [16,25]. Other indicators linked to tumor invasion distance were also proposed, such as the distance from the tumor to the waist of the uterine contour [16] and the distance between the tumor’s deepest invasion point to the cervical stromal ring [17]. The findings of the above studies indicated that the tumor invasion distance was associated with prognosis in CC patients who received surgery. Meanwhile, tumor morphology was also described and found to be associated with prognosis in CC patients who received RT [13]. Nevertheless, few studies have explored the correlation between the invasion distance and prognosis in CC patients without surgery opportunities. Therefore, we proposed a novel definition of invasion distance based on MRI images to facilitate clinicians in prognosis prediction for CC patients who have received RT. According to our analysis, TID could indicate the tumor eccentric growth pattern and the tumor burden. Moreover, the tumor invasion distance was also an independent prognostic factor for OS, PFS, LRFS, and DMFS for CC patients treated with RT. Additionally, for surgical patients, a longer TID could also predict the increasing possibility of parametrium infiltration, adhesion to the posterior wall of the bladder or the anterior wall of the rectum, heightening surgical complexity. Therefore, a longer TID is more indicative of the severity of the condition than the tumor size. It also suggested that patients with a longer TID should transfer surgery to definitive RT promptly. More research is needed to compare the prognostic value between TID and tumor size, and validate the correlation between TID and surgery outcomes in CC.

Except for TID, vaginal invasion and the RT mode were crucial prognostic factors, aligning with findings from previous studies [29,30]. For cases involving the lower 1/3 vagina, comprehensive RT should encompass bilateral groin irradiation [29]. The reported accuracy of MRI in determining vaginal invasion ranged from 83 to 94% [31], although potential inaccuracies due to vaginal wall edema may be addressed by incorporating a biopsy. Despite such challenges, MRI remains valuable in delineating the extent of the tumor, especially when it affects the outer layer of the vagina, leading to adhesion to surrounding tissue or obstruction of the vagina. Regarding the RT mode, concurrent chemotherapy is recommended, but its adoption may be influenced by factors such as age and renal function concerns, etc. [32]. Unfortunately, few studies have evaluated the risk associated with the lack of chemotherapy in patients undergoing RT. In this study, we revealed that the absence of chemotherapy posed a risk comparable to vaginal invasion or ID, which was similar to previous studies. Gurram found that the absence of chemotherapy carried a risk as high as the post-treatment persistent disease for overall survival [32]. Similarly, in one of our previous studies, we also found the lack of concurrent chemotherapy posed a risk equivalent to other factors, resulting in early metastasis in CC patients treated with RT [33].

This study has several strengths. First, to the best of our knowledge, it is the first study to propose TID based on MRI as an independent prognostic factor for CC patients who had undergone RT. The tumor involvement features based on the MRI could standardize and improve the consistency and repeatability of diagnosis. Second, we provided a new risk stratification that improved the discriminability and monotonicity compared to the current 2018 FIGO stage. The new category could give clinicians more prognostic information about CC patients who received RT with regard to individual treatment and surveillance plans.

There are also some limitations to be acknowledged. First, this study was designed retrospectively and selection bias could not be avoided. More multi-center research with larger samples will be needed to validate the reliability of the results. Second, the significance of TID should be further investigated in IIIC-IV stage patients. Third, due to incomplete data, we were unable to include other information like treatment response, Human Papillomavirus (HPV) infection and potentially relevant biomarkers such as the squamous cell carcinoma antigen (SCC-Ag) and carcinoembryonic antigen (CEA), which may have provided additional prognostic insights.

## 5. Conclusions

This study defines TID based on MRI as a novel prognostic factor in CC patients primarily treated with RT. A new risk stratification based on TID showed better discriminability and monotonicity compared to the current 2018 FIGO staging system. The new category could help clinicians stratify high-risk CC patients who need individual additional treatment and intensive follow-up plans.

## Figures and Tables

**Table 1:** Baseline characteristics of patients.

Characteristic	Level	Number of Patients (%)
Total number		218
Age		25–84 (median = 59)
Age (years)	<73	192 (88.1)
	≥73	26 (11.9)
Stage	I	3(1.4)
	II	111(50.9)
	III	104(47.7)
Pathology	NSCC	14 (6.4)
	SCC	204 (93.6)
Invasion distance (median [IQR])		2.71 [2.11, 3.55]
Invasion distance (cm)	<3.9	181 (83.0)
	≥3.9	37 (17.0)
Uterus invasion	no	113 (51.8)
	yes	105 (48.2)
Vaginal involvement	upper2/3	193 (88.5)
	low1/3	25 (11.5)
Hydronephrosis	no	213 (97.7)
	yes	5 (2.3)
Treatment	CCRT	159 (72.9)
	RT	59 (27.1)

NSCC: Non-squamous cervical cancer, SCC: squamous cervical cancer, IQR: Interquartile Range, CCRT: concurrent chemoradiotherapy, RT: radiotherapy.

**Table 2:** Univariate and multivariate analysis of prognostic factors for OS, PFS, LRFS, and DMFS.

Items	OS				PFS			
	Univarite		Multivariate		Univarite		Multivariate	
	HR (95% CI)	*p*	HR (95% CI)	*p*	HR (95% CI)	*p*	HR (95% CI)	*p*
Age								
≥73 vs. <73	3.82 (1.67–8.28)	0.001	1.38 (0.58–3.29)	0.466	2.5 (1.28–4.88)	0.007	1.1 (0.51–2.36)	0.800
Histology								
SCC VS NSCC	2.23 (0.3–16.33)	0.430			1.16 (0.36–3.74)	0.798		
Treatment								
CCRT vs. RT	0.28 (0.14–0.56)	<0.001	0.32 (0.15–0.7)	0.004	0.39 (0.23–0.68)	0.001	0.41 (0.22–0.77)	0.005
Vaginal invasion								
low1/3 vs. upper 2/3	4.64 (2.2–9.82)	<0.001	2.58 (1.16–5.76)	0.021	3.12 (1.63–5.96)	0.001	2.02 (1.01–4.04)	0.048
Uterus involvement								
Yes vs. No	0.72 (0.36–1.46)	0.367			0.88 (0.51–1.53)	0.648		
TID								
≥3.9 vs. <3.9	3.42 (1.67–7)	0.001	3.00 (1.41–6.38)	0.004	2.49 (1.36–4.55)	0.003	2.37 (1.25–4.49)	0.008
Hydronephrosis								
Yes vs. No	1.26 (0.17–9.25)	0.819			0.73 (0.1–5.32)	0.76		
**Items**	**DMFS**				**LRFS**			
	**Univarite**		**Multivariate**		**Univarite**		**Multivariate**	
	**HR (95% CI)**	***p***	**HR (95% CI)**	***p***	**HR (95% CI)**	***p***	**HR (95% CI)**	***p***
Age								
≥73 vs. <73	0.94 (0.29–3.11)	0.923			2.49 (0.82–7.57)	0.109		
Histology								
SCC VS NSCC	0.66 (0.2–2.19)	0.502			27051606.71 (0–Inf)	0.997		
Treatment								
CCRT vs. RT	0.7 (0.33–1.49)	0.355			0.33 (0.13–0.83)	0.018	0.29 (0.12–0.75)	**0.01**
Vaginal invasion								
Low 1/3 vs. upper 2/3	1.9 (0.73–4.96)	0.188			2.68 (0.88–8.15)	0.083		
Uterus involvement								
Yes vs. No	0.57 (0.28–1.2)	0.140			2.19 (0.82–5.83)	0.118		
TID								
≥3.9 vs. <3.9	2.45 (1.13–5.31)	0.024	2.45 (1.12–5.32)	0.024	3.5 (1.36–9.04)	0.010	3.94 (1.52–10.24)	0.005
Hydronephrosis								
Yes vs. No	0 (0–Inf)	0.996			0 (0–Inf)	0.997		

**Table 3:** Discriminatory ability of the new risk stratification and 2018 FIGO stage system for 3-year OS among CC patients with RT.

System	χ^2^ Test or Linear Trend	AIC	C Statistic
New risk stratification	28.03	314	0.74
2018 FIGO stage system	9.35	331	0.646

## Data Availability

The data used or analyzed of the current study is available from the corresponding author on reasonable request.

## Supplementary Materials

The following supporting information can be downloaded at: https://www.mdpi.com/article/10.3390/curroncol32060355/s1, Figure S1: Patient selection.
