# Supplementary figures and images for: Tumor Invasion Distance Based on MRI Is a Novel Prognostic Indicator for I-IIIB Cervical Cancer Patients Treated with Radiotherapy
Source: Curr Oncol. 2025 Jun 16;32(6):355. doi: 10.3390/curroncol32060355 (PMC12192517; doi:10.3390/curroncol32060355)

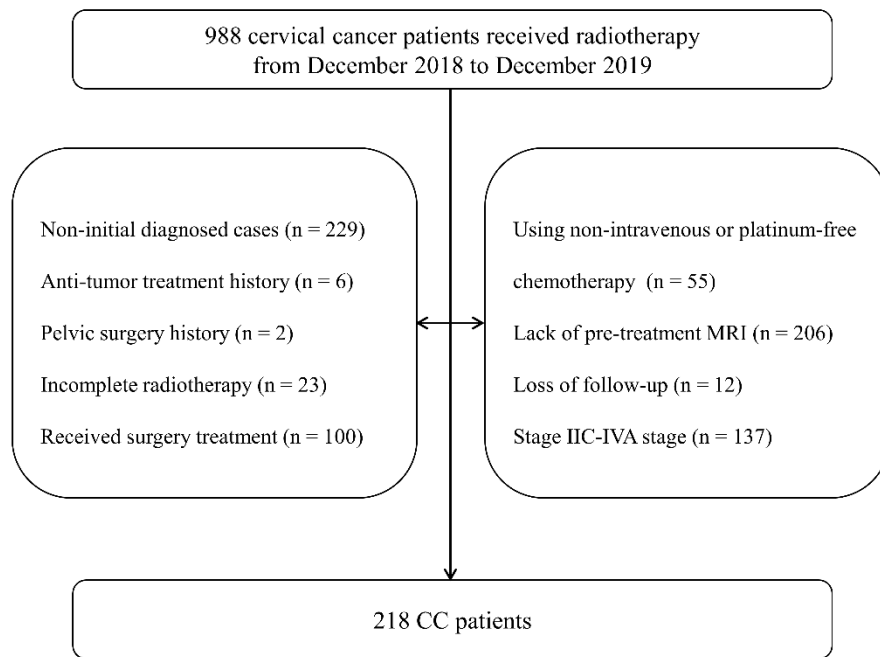

Figure S1. Patient selection.

Supplement: Supplementary file 1 [file curroncol-32-00355-s001.zip › curroncol-3625557-supplementary.pdf]
